# The mediating effect of psychological capital in the relationship between stress and college adjustment among nursing students in South Korea: a cross-sectional study

**DOI:** 10.7586/jkbns.25.021

**Published:** 2025-05-22

**Authors:** Yujin Jeong, Joohee Shim

**Affiliations:** College of Nursing, Yeungnam University College, Daegu, Korea

**Keywords:** Students, nursing, Psychology, positive, Stress, psychological

## Abstract

**Purpose:**

The aim of this study was to identify the mediating effect of psychological capital on the relationship between stress and college adjustment in nursing students.

**Methods:**

This study was conducted using a descriptive, cross-sectional design. A sample of 284 nursing students was recruited from various South Korean universities. Data were collected through an online survey conducted from October 2021 to February 2022. The questionnaire used in this study included the Revised Life Stress Scale for College Students, the Psychological Capital Scale, the College Adjustment Scale, and general characteristics of nursing students. Data analysis was conducted using SPSS version 25.0 and PROCESS macro version 4.1.

**Results:**

Stress exhibited significant direct and total effects on college adjustment. Additionally, psychological capital, the mediating variable, had a significant direct effect on college adjustment. Moreover, the indirect effect of stress on college adjustment through psychological capital was significant.

**Conclusion:**

It is necessary to develop strategies to strengthen nursing students’ psychological capital to reduce stress and facilitate successful college adjustment.

## INTRODUCTION

Recently, in response to the shortage of nurses in medical institutions, the enrollment quota for nursing colleges in the South Korea is continuously expanding [1]. In addition to implementing this expansion policy, it is also important for nursing students to successfully adapt to college life without dropping out, to enable them to develop their competencies as professional nurses [2]. Nursing education aims for students to develop theoretical knowledge and clinical skills for professional nursing roles [3]. Given its academic nature, nursing students adapt to not only difficult specialized knowledge but also on-campus and clinical practice [4]. Consequently, nursing students can have difficulties adjusting to college life, which may result in them dropping out [5]. The dropout rate among nursing students is influenced by complex interactions of various elements such as personal, institutional, political, and professional factors [6]. Furthermore, dropout occurs not only in the early stages but also later stages in their university education [7,8]. Therefore, it is essential to explore effective strategies that help nursing students adapt well throughout their college years and successfully complete their studies.

Stress is defined as an individual’s perception that external demands exceed their ability to cope with those demands [9]. Prolonged exposure to stress can impair autonomic nervous system function, thereby disrupting the homeostatic balance of the human body [10]. Psychologically, stress can lead to depression, emotional instability, and anger, potentially progressing to depressive disorders or other psychiatric symptoms [11,12]. Nursing students commonly face significant stress, which is recognized as a global issue [13]. Those engaged in both academic coursework and clinical practice often report experiencing higher stress levels compared to students in other undergraduate programs [14]. Thus, various negative life events that nursing students experience during their college life may add to their stress. Nursing students experience psychological distress due to negative life events such as academic pressure and interpersonal relationships [15]. Stress during the academic process can negatively impact various academic outcomes, including motivation, achievement, and college adaptation [16-18], potentially reducing the number of nursing students who successfully graduate. Therefore, it is essential to explore ways to reduce stress among nursing students to have them adapt well in college life.

Psychological capital (PsyCap) may serve as a crucial factor in supporting nursing students' adaptation to college life and alleviating stress. PsyCap is a higher-order construct comprising four positive psychological resources: self-efficacy, hope, resilience, and optimism [19]. These components are theoretically interrelated; for instance, optimistic individuals tend to expect positive outcomes, self-efficacious individuals are more likely to set and achieve challenging goals, hopeful individuals pursue multiple pathways toward their goals, and resilient individuals are able to recover from adversity when encountering obstacles [20]. Collectively, these psychological resources foster internalized control and goal-directed behavior, thereby promoting psychological well-being even in the face of stressors [9,10]. Furthermore, emerging evidence suggests that PsyCap may also be associated with physiological responses to stress, including cortisol regulation, cardiovascular recovery, and immune function [21]. PsyCap is well-recognized for its significant role in the work environment, positively impacting job related outcomes such as work attitude, performance, and job satisfaction [22]. Furthermore, previous studies have highlighted the relevance of PsyCap to various academic outcomes among university students, such as performance, participation, burnout, adaptation, stress, and motivation [23].

To date, research on PsyCap has primarily focused on workers, including clinical nurses, examining its associations with key occupational outcomes such as stress, intention to leave, and job satisfaction [24]. Meanwhile, interest in PsyCap has also been growing among college and nursing student populations, with studies reporting its associations with various academic and psychological factors [18,25]. Similarly, several studies conducted in South Korea have examined the relationships between PsyCap and key variables among nursing students, including stress [26], academic achievement [27], academic burnout [28], and college life adaptation [29]. To date, few studies in Korea have specifically examined the mediating role of PsyCap in the relationship between stress and college adjustment among nursing students. Although a previous study conducted in China [18] confirmed this mediating effect, it is necessary to re-examine the relationship in the Korean context, given cultural and educational differences. Considering these points, PsyCap may play an important role in alleviating the stress nursing students experience due to negative life events during college and in supporting their successful adaptation to college life. Therefore, this study aims to fill a significant gap in the literature by examining the mediating effect of PsyCap on the relationship between stress and college adjustment among Korean nursing students.

## METHODS

### 1. Study design

This study, which targeted nursing students, employed a descriptive cross-sectional design to examine how stress and PsyCap are related to college life adjustment. In addition, it explored whether PsyCap functions as a mediator in the relationship between stress and students’ adjustment to university life.

### 2. Participants

Undergraduate nursing students from various regions of South Korea—including Seoul and the provinces of Gyeonggi, Gangwon, Chungcheong, Jeolla, and Gyeongsang—were recruited for this study. Participants were eligible to take part if they met the following inclusion criteria: (1) were undergraduate nursing students aged 18 years or older, (2) were currently enrolled in the second to fourth year of a four-year bachelor's program in nursing, and (3) understood the purpose of the study and voluntarily agreed to participate.

A total of 309 responses were initially collected. After excluding 25 participants who did not meet the inclusion criteria, 284 valid responses were included in the final analysis. This sample size met the recommended range (115~285 participants) for mediation analysis to detect an indirect effect with a power of .80 [30].

### 3. Instruments

#### 1) General characteristics

General characteristics were assessed using seven questions about age, gender, grade, motivation for applying to nursing department, satisfaction with their major in nursing, health status, and interpersonal relationships.

#### 2) Stress

Stress was assessed using a revised version of the Life Stress Scale designed for college students, originally developed and validated by Chon et al. [31]. This scale assesses the frequency of stressful events experienced by college students over the past year, as well as the importance of each event if experienced more than once. This scale comprises 50 items divided into two dimensions: interpersonal relationships and task-related stress. The interpersonal relationship dimension consists of four areas: lover, friend, family, and faculty. The task-related stress dimension consists of four areas: grade, economy, future, and value.

In this study, only the occurrence of each stress event was assessed. The stress scale utilizes a 4-point Likert system, with higher scores reflecting greater frequency of stressful events. The Cronbach's α coefficients of the life stress scale at the time of development ranged from .75 to .88 [31]. In the present study, the coefficients for the total life stress scale, interpersonal relationship dimension, and task-related stress dimension were .96, .96, and .92, respectively.

#### 3) PsyCap

A validated Korean adaptation of the instrument, originally developed by Luthans et al. [19], was used to assess PsyCap, as modified and validated for the Korean context by Lim [32]. This scale comprises 18 items divided into four components: optimism, self-efficacy, hope, and resilience. The measurement of responses utilized a 5-point Likert scale, with higher aggregate scores reflecting increased levels of PsyCap. In the original study by Lim [32], the Cronbach's α was reported as .93, indicating excellent reliability. The current investigation yielded a Cronbach's α of .89, further supporting the scale's strong internal consistency in this sample of nursing students.

#### 4) College adjustment

To assess nursing students’ college adjustment, we employed the scale developed by Jeong and Park [33]. This scale comprises 19 items and five constructs: academic activities, interpersonal relations, personal psychology, social participation, and career preparation. A 5-point Likert scale was used for each item, with greater total scores reflecting better adjustment to college life. The scale used in this study showed a statistically significant correlation (.59) with the existing college adjustment scale by Baker and Siryk [34] and was verified to be highly valid. The Cronbach’s α of the scale was .86 at the time of its development, and .85 in the present study.

### 4. Data collection

Data were collected between October 2021 and February 2022 through the “Nurse Preparation Group,” an online community specifically for nursing students. Recruitment materials included a Google survey link and a clear explanation of the study’s purpose, methodology, and inclusion criteria. Only students who voluntarily consented after reviewing the study details were granted access to the survey. To ensure proper participation, both inclusion and exclusion criteria were clearly stated in advance. The online questionnaire consisted of sections on general characteristics and stress (50 items), PsyCap (18 items), and college adjustment (19 items).

### 5. Data analysis

Statistical analyses were performed using SPSS version 25.0 (IBM Corp., Armonk, NY, USA) and the PROCESS macro version 4.1. Prior to data analysis, a process of data cleaning and screening was conducted, and no missing values were found across all scales. Descriptive statistics – including frequencies, proportions, means, and standard deviations (SDs) – were employed to summarize the general characteristics of the nursing students. Participant stress, PsyCap, and college adjustment were presented as means ± SD. Group differences in stress, PsyCap, and college adjustment were tested using one-way analysis of variance (ANOVA) and Student’s t-tests, with Tukey's Honestly Significant Difference applied for post hoc comparisons where appropriate. In cases where the assumption of equal variances was not met, group means were compared using Welch’s ANOVA, and post hoc analyses were conducted with Dunnett’s T3. The associations between stress, PsyCap, and college adjustment were analyzed using bivariate correlation coefficients.

Model 4 of the SPSS PROCESS macro [35] was employed to examine the mediating role of PsyCap. In the model, age, gender, satisfaction with nursing major, health status, and interpersonal relationships were included as covariates. The indirect effect was evaluated using 5,000 bootstrap samples, and bias-adjusted 95% confidence intervals were estimated to determine significance.

### 6. Ethical considerations

This study adhered to the ethical standards of the Declaration of Helsinki. Ethical approval was obtained from the Institutional Review Board (IRB) of Yeungnam University College in Korea prior to data collection (IRB No. 2-7008156-AB-N-01-A-2021-002). All nursing students were provided with detailed information about the study and participated voluntarily. A study information page describing the purpose, procedures, and ethical considerations was displayed at the beginning of the online survey. Participants who clicked the “Agree” button were automatically directed to the questionnaire. Informed consent was thus obtained prior to participation. Anonymity and confidentiality were ensured, and all data were treated as strictly confidential. Participants’ phone numbers were collected solely for the purpose of providing compensation. To prevent duplicate participation, the numbers were verified for uniqueness and permanently deleted after compensation was provided.

## RESULTS

### 1. Participant characteristics

Of the 284 nursing students who participated in this study, the majority were women (90.8%), with a mean age 22.98 years (SD 2.96). Fourth-year students accounted for the majority (46.8%), followed by third-year (30.3%), and second-year (22.9%) students. Regarding the motivation for applying to the nursing department, the reason with the highest response rate was high employment rate after graduation (50.0%), followed by own desire (28.9%), family and others’ recommendations (13.0%), and high school grades (8.1%). Regarding satisfaction with their major in nursing, the average score was 3.62 on a 5-point Likert scale. In terms of health status, students rated their health at an average of 3.58, while interpersonal relationships were rated higher at 3.74 (Table 1).

### 2. Participant stress, PsyCap, and college adjustment

The average stress score among the nursing students was 0.93 ± 0.56 (range: 0~3). Notably, the level of task-related stress (1.13 ± 0.53) was considerably higher than that of interpersonal stress (0.69 ± 0.65), suggesting that academic-related demands may be a more prominent source of stress than interpersonal relationships among the participants.

The overall mean score for PsyCap was 3.42 ± 0.56 (range: 1~5), with comparable levels observed across its four subcomponents: self-efficacy (3.39 ± 0.63), optimism (3.45 ± 0.67), hope (3.45 ± 0.67), and resilience (3.37 ± 0.82). These findings indicate that the participants possessed relatively balanced psychological resources.

The average score for college adjustment was 65.62 ± 10.41 (range: 19~95), indicating a generally moderate to high level of adjustment among the participants (Table 2).

### 3. Differences in stress, PsyCap, and college adjustment according to general characteristics

Table 3 presents the differences in stress, PsyCap, and college adjustment based on nursing students’ demographic and academic characteristics.

Stress was positively correlated with age (r = .29, *p* < .001), indicating that older students tended to experience stress more frequently. Moreover, female students had a higher frequency of stress than male students (t = −2.53, *p* = .012), suggesting that gender may influence how frequently stress is experienced. In addition, there was a difference in the frequency of experiencing stress according to grade level (F = 3.93, *p* = .022), with second-year students reporting higher stress compared to other grades.

Those who applied because of the high employment rate experienced more stress than those who applied voluntarily (F = 2.70, *p* = .046). The higher the satisfaction with nursing major, the lower the stress (r = − .30, *p* < .001); the better the interpersonal relationship, the lower the stress caused by college life (r = − .32, *p* < .001). These findings suggest that both academic satisfaction and positive interpersonal relationships may serve as protective factors against stress.

Furthermore, the results showed that the higher the satisfaction with nursing major, the higher the PsyCap (r = .39, *p* < .001) and college adjustment (r = .48, *p* < .001). Interpersonal relationships were positively and significantly correlated with PsyCap (r = .41, *p* < .001) and college adjustment (r = .45, *p* < .001), suggesting that interpersonal relationships may contribute to psychological resources and successful adaptation to college life.

### 4. Correlations among stress, PsyCap, and college adjustment

Stress was negatively correlated with PsyCap (r = − .40, *p* < .001) and college adjustment (r = − .41, *p* < .001), indicating that students who experienced stress more frequently tended to have lower psychological resources and poorer adaptation to college. Conversely, higher levels of PsyCap were significantly associated with better college adjustment (r = .71, *p* < .001), suggesting that PsyCap may play a central role in supporting students’ adjustment process (Table 4).

### 5. Testing the mediating role of PsyCap

**Figure 1.**
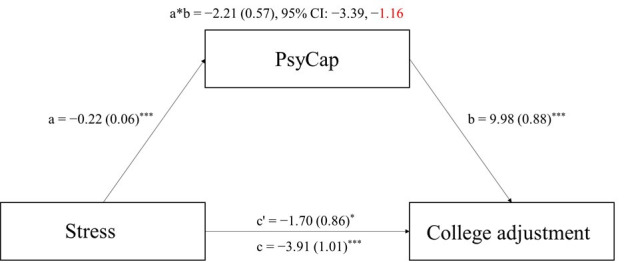
Model examining the mediating effect of PsyCap in the relationship between stress and college adjustment. PsyCap = Psychological capital. The parameter estimates are presented as B (standard error). a = direct effect of stress on PsyCap; b = direct effect of PsyCap on college adjustment; c’ = direct effect of stress on college adjustment; c = total effect of stress on college adjustment; a*b = indirect effect of stress on college adjustment through PsyCap. CI = Confidence interval; *p < 0.05; **p < 0.01; ***p < 0.001.

Figure 1 shows the results of the mediation analysis, which explored whether PsyCap serves as a mediator between stress and college adjustment. The results revealed that the total effect of stress on college adjustment was significant (B = −3.91, standard error [SE] = 1.01, 95% CI: −5.91, to −1.92; R^2^ = 0.41). In addition, stress had direct effects on PsyCap (B = −0.22, SE = 0.06, 95% CI: −0.33, to −0.11) and college adjustment (B = −1.70, SE = 0.86, 95% CI: −3.39, to −0.01). PsyCap had a direct effect on college adjustment (B = 9.98, SE = 0.88, 95% CI: 8.25, to 11.71). Furthermore, stress had a significant indirect effect on college adjustment through PsyCap (B = −2.21, SE = 0.57, 95% CI: −3.39, to −1.16).

## DISCUSSION

This study explored the associations among stress, PsyCap, and college adjustment, and assessed whether PsyCap mediated the relationship between stress and college adjustment in nursing students. The main results indicated that PsyCap plays a mediating role between stress and college adjustment. Furthermore, associations between stress, PsyCap, and college adjustment were identified.

Our findings confirmed that PsyCap significantly mediates the relationship between stress and college adjustment. PsyCap, which is a multi-dimensional construct, comprises four key components (hope, resilience, efficacy, and optimism). Prior research suggests that PsyCap is positively associated with school-related outcomes, including college adjustment and academic performance, and is inversely related to stress among university students [18,25]. In other words, PsyCap can positively influence individual adaptation to stressful events and act as a buffer, reducing the impact of stress on negative outcomes [18,36]. PsyCap is considered a state-like resource that can develop and change over time [20]. Thus, nursing educators should seek ways to enhance PsyCap among nursing students to effectively reduce stress and promote successful college adjustment. To enhance student PsyCap, universities can provide training programs that focus on fostering self-efficacy, optimism, hope, and resilience. These programs can be effectively delivered through various methods, including seminars, group counseling sessions, or integration into the formal curriculum [37].

Although PsyCap was treated as a single construct in the main analysis, an additional analysis was conducted to examine the relative contribution of each subcomponent. The results showed that hope exhibited the strongest mediating effect between stress and college adjustment, followed by self-efficacy and optimism, while the effect of resilience was not statistically significant. These findings suggest that hope, which facilitates the pursuit of multiple pathways toward meaningful goals and sustains motivation, may serve as a particularly important psychological resource for adapting to college life under stress [20]. Therefore, interventions that specifically target the development of hope may be especially beneficial in supporting nursing students’ adaptation to academic and clinical challenges. Additional results showed that the participants’ interpersonal relationships were negatively correlated with stress and positively correlated with both PsyCap and college adjustment. The quality of individual relationships with others is linked to the support they receive from these relationships, which is commonly referred to as social support [38]. Support from peers and faculty can play a significant role in reducing stress and improving positive academic outcomes in students [39,40]. Moreover, according to previous findings, social support has a positive relationship with PsyCap, suggesting that social support can serve as a significant factor in enhancing individual PsyCap [20,41,42]. Therefore, providing adequate support to nursing students is important to reduce stress, enhance PsyCap, and promote college adjustment. Particularly, in undergraduate nursing programs, student-faculty relationships are regarded as central factors contributing to student academic success [39]. Thus, the faculty should assist students by understanding their needs and providing the necessary support to facilitate development and learning. Additionally, faculty members can provide support to nursing students by implementing a peer mentoring program that effectively reduces stress and promotes successful college adjustment [43,44]. Clinical practice, an essential part of nursing education, is widely recognized as a major source of stress among nursing students [45,46]. Therefore, it is necessary to help students cope with stress in clinical practice by creating collaborative environment among students, faculty, and clinical staff.

Nonetheless, a notable finding in the present study is that second-year students—prior to participating in hospital-based clinical training—reported more frequent experiences of stress. In Korea, second-year nursing students begin foundational coursework and basic nursing practice, marking the beginning of their major-related education. During this phase, they are exposed to an increased academic workload, which may lead to greater academic pressure. Furthermore, as they learn basic nursing skills, they may become more aware of their limited knowledge and clinical competence, contributing to more frequent stress experiences. Supporting this interpretation, Admi et al. [47] reported that second-year preclinical students experienced significantly higher stress levels, particularly in situations involving inadequate preparation for clinical tasks. These findings suggest that stress management strategies should not be limited to upper-year students engaged in clinical practice but should be introduced from the early stages of nursing education through proactive and collaborative educational support.

In this study, a significant difference in the degree of stress among participants was observed depending on their motivation to apply to nursing department. In addition, our findings showed that the majority of the participants opted for a nursing major based on factors such as employment rate after graduation, family and others’ recommendations, and high school grades. In contrast, approximately 30% of the participants responded that they chose nursing major based on their desires. In the South Korea, college students often choose a major according to their high school grades or parent expectations rather than choosing a major considering their aptitude and interest. Previous studies have also reported that more nursing students applied to the nursing department because of the high employment rate after graduation or the recommendations from others, rather than their own aptitude [48,49].

Due to the academic nature of nursing, nursing students have been reported to experience higher levels of stress than students in other majors [14]. In this context, students who apply to the nursing department without understanding their aptitudes and majors are likely to experience more stress during their academic journey. Therefore, it is important to consider these points when developing stress management plans for nursing students. Nursing educators should provide various career information related to nursing, in addition to the clinical field, so that nursing students can find a connection between their aptitude and their major [48]. In addition, it is necessary to provide services that can support stress management and department adaptation for nursing students as well as to establish courses related to learning methods and various academic counseling services [50].

The present findings confirm that PsyCap plays a mediating role in the relationship between stress and college adjustment. These results are expected to substantially contribute to the establishment of strategies aimed at reducing stress and fostering adaptation to college life among nursing students in the future. However, it is important to acknowledge the limitations of this study. First, participants were recruited through convenience sampling; thus, the results should be generalized with caution. Given the cross-sectional nature of this study, caution is warranted when interpreting causal relationships among the variables.

To advance this line of research, future investigations may consider multidimensional approaches to deepen the understanding of how PsyCap operates in the context of stress. Specifically, incorporating physiological indicators such as cortisol levels, heart rate variability, or immune biomarkers may help clarify the mechanisms linking PsyCap and stress. Such an approach would enhance understanding of how PsyCap functions within biopsychosocial stress responses and may contribute to expanding its relevance in the field of biological nursing.

## CONCLUSION

Our research revealed that PsyCap plays an intermediary role in the connection between stress levels and university adaptation among students in nursing programs. This investigation uncovered the significant mediating influence of positive psychological resources on how nursing students manage stress and adjust to college life. Stress reduces PsyCap, which has a negative indirect effect on college adaptation. However, PsyCap acts as a parameter to strengthen college adaptation, improving college students' ability to adapt to college life and reducing the negative effects of stress. Given its multidimensional nature, encompassing efficacy, hope, resilience, and optimism, PsyCap is a valuable resource that can be cultivated to enhance student well-being and academic success. Therefore, to facilitate successful college adjustment and to support academic achievement among nursing students, it is necessary to implement training programs that enhance PsyCap.

## Figures and Tables

**Table 1.** Sociodemographic and Academic Characteristics of Nursing Students (N = 284)

Characteristics	n (%)	M ± SD
Age (yr)		22.98 ± 2.96
Sex		
Men	26 (9.2)	
Women	258 (90.8)	
Year in school		
2	65 (22.9)	
3	86 (30.3)	
4	133 (46.8)	
Motivation for applying to the nursing department		
High school grades	23 (8.1)	
Own desire	82 (28.9)	
Highest employment rate after graduation	142 (50.0)	
Family and others’ recommendation	37 (13.0)	
Satisfaction with nursing major		3.62 ± 0.80
Health status		3.58 ± 0.78
Interpersonal relationship		3.74 ± 0.80

M = Mean; SD = Standard deviation.

**Table 2.** Degrees of Stress, PsyCap, and College Adjustment of Participants (N = 284)

Variables	M ± SD	Min	Max	Range
Stress				
Total	0.93 ± 0.56	0	1.90	0~3
Interpersonal dimension	0.69 ± 0.65	0	2.00	0~3
Task-related stress	1.13 ± 0.53	0	1.96	0~3
PsyCap				
Total	3.42 ± 0.56	1.33	4.94	1~5
Self-efficacy	3.39 ± 0.63	1.00	5.00	1~5
Optimism	3.45 ± 0.67	1.40	5.00	1~5
Hope	3.45 ± 0.67	1.00	5.00	1~5
Resilience	3.37 ± 0.82	1.00	5.00	1~5
College adjustment				
Total	65.62 ± 10.41	37.00	95.00	19~95
Academic activities	15.02 ± 2.86	7.00	20.00	4~20
Career preparation	13.59 ± 3.10	4.00	20.00	4~20
Personal psychology	14.67 ± 2.97	6.00	20.00	4~20
Interpersonal relations	12.45 ± 3.38	4.00	20.00	4~20
Social participation	9.89 ± 2.45	3.00	15.00	3~15

PsyCap = Psychological capital; M = Mean; SD = Standard deviation; Min = Minimum; Max = Maximum.

**Table 3.** Differences in Stress, PsyCap, and College Adjustment According to Participants’ Characteristics (N = 284)

Characteristics	n	Stress	PsyCap	College adjustment
		M ± SD	M ± SD	M ± SD
Age (yr)	284			
r (*p)*		0.29 (< .001)	−0.05 (.366)	−0.08 (.158)
Sex				
Men	26	0.66 ± 0.50	3.40 ± 0.63	66.54 ± 11.88
Women	258	0.95 ± 0.56	3.42 ± 0.55	65.52 ± 10.27
t (*p)*		−2.53 (.012)	−0.14 (.889)	0.47 (.636)
Year in school				
2^a^	65	1.10 ± 0.59	3.38 ± 0.60	64.65 ± 11.03
3^b^	86	0.87 ± 0.50	3.37 ± 0.49	63.97 ± 9.58
4^c^	133	0.87 ± 0.56	3.47 ± 0.58	67.16 ± 10.48
F (*p)*		3.93 (.022)^†^	1.19 (.305)	2.86 (.059)
		a > b,c^‡^		
Motivation for applying to the nursing department				
High school grades^a^	23	0.94 ± 0.59	3.39 ± 0.62	67.09 ± 12.34
Own desire^b^	82	0.78 ± 0.57	3.56 ± 0.59	67.50 ± 10.03
Highest employment rate after graduation^c^	142	1.00 ± 0.55	3.36 ± 0.53	64.45 ± 9.74
Family and others’ recommendation^d^	37	0.96 ± 0.52	3.32 ± 0.51	65.00 ± 12.10
F (*p)*		2.70 (.046)	2.74 (.044)	1.70 (.168)
		b < c	b > c	
Satisfaction with nursing major	284			
r (*p)*		−0.30 (< .001)	0.39 (< .001)	0.48 (< .001)
Health status	284			
r (*p)*		−0.39 (< .001)	0.37 (< .001)	0.31 (< .001)
Interpersonal relationship	284			
r (*p)*		−0.32 (< .001)	0.41 (< .001)	0.45 (< .001)

PsyCap = Psychological capital; SD = Standard deviation.

^†^Welch analysis of variance; ^‡^Dunnett’s T3 post-hoc test.

**Table 4.** Correlations among Study Variables (N = 284)

Variables	Stress	PsyCap	College adjustment
	r (*p*)		
Stress	1	−.40 (< .001)	−.41 (< .001)
PsyCap	-	1	.71 (< .001)

PsyCap = Psychological capital.

## Data Availability

The dataset supporting the conclusions is available from the corresponding author on reasonable request.
